# Supplementary material for: Surface Microbiome Profiling of Dental Elevators Using Third‐Generation Sequencing: Implications for Infection Control in Dental Practice
Source: Microbiologyopen. 2025 Dec 8;14(6):e70178. doi: 10.1002/mbo3.70178 (PMC12685768; doi:10.1002/mbo3.70178)
Supplement: Supplementary file 1 — customer_backup. [file MBO3-14-e70178-s001.zip › customer_backup/customer_backup/taxa_summary/heatmap/treat/treat.genus.reabundance.heatmap.pdf]

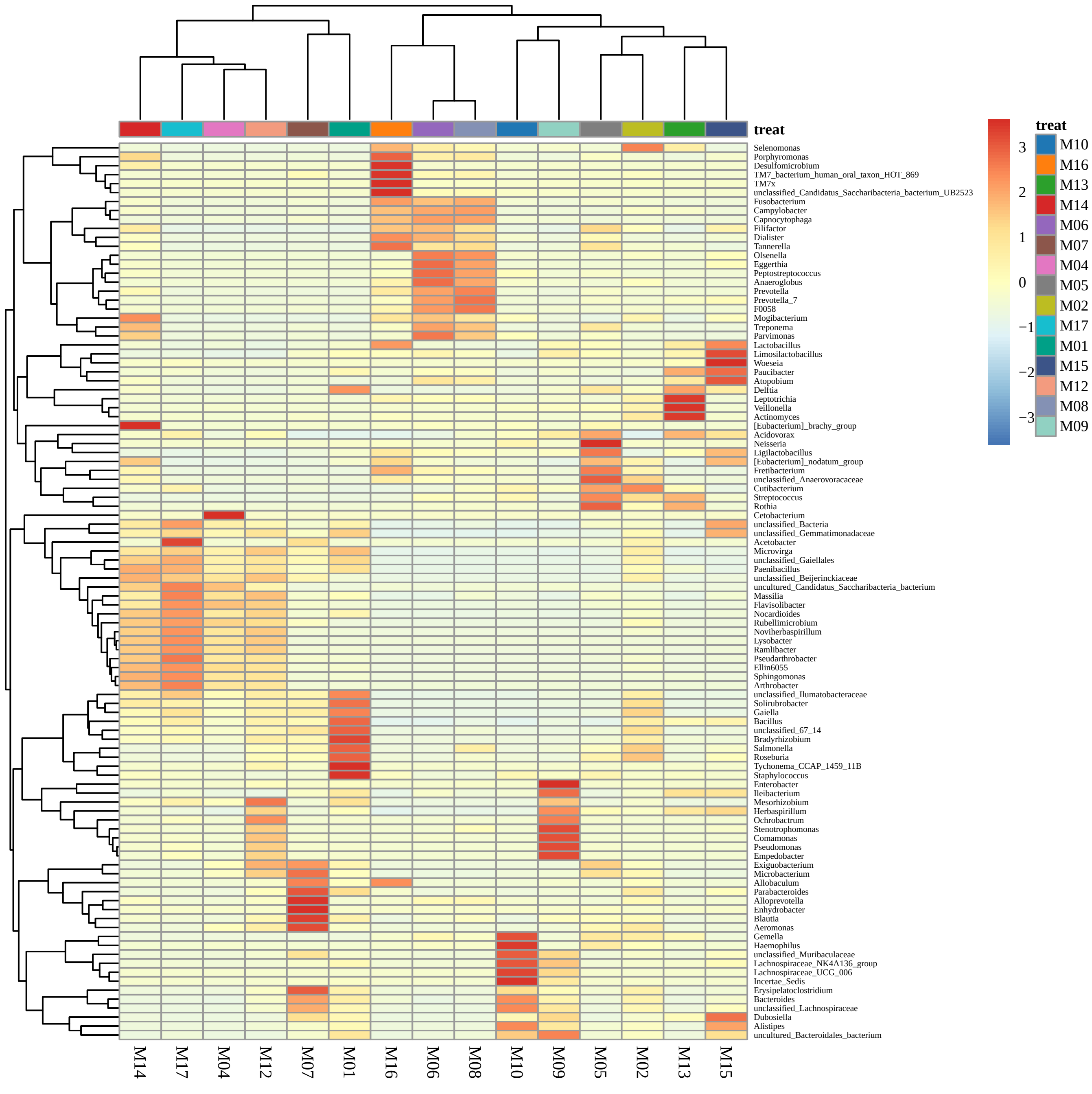

treat
Selenomonas
Porphyromonas
Desulfomicrobium
TM7_bacterium_human_oral_taxon_HOT_869
TM7x
unclassified_Candidatus_Saccharibacteria_bacterium_UB2523
Fusobacterium
Campylobacter
Capnocytophaga
Filifactor
Dialister
Tannerella
Olsenella
Eggerthia
Peptostreptococcus
Anaeroglobus
Prevotella
Prevotella_7
F0058
Mogibacterium
Treponema
Parvimonas
Lactobacillus
Limosilactobacillus
Woeseia
Paucibacter
Atopobium
Delftia
Leptotrichia
Veillonella
Actinomyces
[Eubacterium]_brachy_group
Acidovorax
Neisseria
Ligilactobacillus
[Eubacterium]_nodatum_group
Fretibacterium
unclassified_Anaerovoracaceae
Cutibacterium
Streptococcus
Rothia
Cetobacterium
unclassified_Bacteria
unclassified_Gemmatimonadaceae
Acetobacter
Microvirga
unclassified_Gaiellales
Paenibacillus
unclassified_Beijerinckiaceae
uncultured_Candidatus_Saccharibacteria_bacterium
Massilia
Flavisolibacter
Nocardioides
Rubellimicrobium
Noviherbaspirillum
Lysobacter
Ramlibacter
Pseudarthrobacter
Ellin6055
Sphingomonas
Arthrobacter
unclassified_Ilumatobacteraceae
Solirubrobacter
Gaiella
Bacillus
unclassified_67_14
Bradyrhizobium
Salmonella
Roseburia
Tychonema_CCAP_1459_11B
Staphylococcus
Enterobacter
Ileibacterium
Mesorhizobium
Herbaspirillum
Ochrobactrum
Stenotrophomonas
Comamonas
Pseudomonas
Empedobacter
Exiguobacterium
Microbacterium
Allobaculum
Parabacteroides
Alloprevotella
Enhydrobacter
Blautia
Aeromonas
Gemella
Haemophilus
unclassified_Muribaculaceae
Lachnospiraceae_NK4A136_group
Lachnospiraceae_UCG_006
Incertae_Sedis
Erysipelatoclostridium
Bacteroides
unclassified_Lachnospiraceae
Dubosiella
Alistipes
uncultured_Bacteroidales_bacterium
M14
M17
M04
M12
M07
M01
M16
M06
M08
M10
M09
M05
M02
M13
M15
3
2
1
0
−1
−2
−3
treat
M10
M16
M13
M14
M06
M07
M04
M05
M02
M17
M01
M15
M12
M08
M09
